# The One Law of Life

**Published:** 1887-01-29

**Authors:** Helen Milman


### THE ONE LAW OF LIFE.

That love is universal—infinite,
Is the one law of life!
The more we love
The more we live, for love is life, is God!
And looking up we see th' eternal light
Shine through the darkness of the sin and strife,
Casting a ray of gladness on a path
O'ertwined with briars, thorns, and ivy boughs,
Which, till the light of heaven pierced the gloom,
Dim'd all the joy of sky, and birds, and spring.
Some serve Him well, who in the din and noise
And centre of the battle-field of life
Work for His sake, in loving tenderness;
In such a service waging war with sin,
Self lost in toil—an ever endless task.
While some, may-be, upon a sick bed lie,
And simply *only suffer* for His sake,
And for no reason but it is His will—
Part of a plan, His own redemption work.
Only to suffer! yet feel Him all in all!
In sonship perfected; by failures tried;
Left bleeding for a time, perchance, yet healed
By the same hand which wounded grievously.
And yet as soldiers still, whose work is great
As those who served Him in an active life:
Yea, even greater, nearer kin to Him
Who came to earth, only to suffer here,
So we in pain might shine for Him, and see
In all one brotherhood, all love! all God!
Tools in our Master's hand; to do His will
In His own way, unmindful of the pain,
If only *used by Him*, to prove the love
He beareth for His loved ones here on earth—
To do His will!—till, there beyond the stars
We wake! in His own likeness satisfied!

Helen Milman.

